# Patterns of intimate partner violence among perinatal women with depression symptoms in Khayelitsha, South Africa: a longitudinal analysis

**DOI:** 10.1017/gmh.2018.1

**Published:** 2018-04-12

**Authors:** M. Schneider, E. Baron, T. Davies, M. Munodawafa, C. Lund

**Affiliations:** 1 Department of Psychiatry and Mental Health, Alan J Flisher Centre for Public Mental Health, University of Cape Town, South Africa; 2 Centre for Global Mental Health, Institute of Psychiatry, Psychology and Neuroscience, King's College London, UK

**Keywords:** IPV, LMICS, longitudinal data, perinatal depression

## Abstract

**Background.:**

A combination of intimate partner violence (IPV) and depression is a common feature of the perinatal period globally. Understanding this association can provide indications of how IPV can be addressed or prevented during pregnancy. This paper aims to determine the prevalence and correlates of IPV among pregnant low-income women with depressive symptoms in Khayelitsha, South Africa, and changes in IPV reports during the course of the perinatal period.

**Methods.:**

This study is a secondary analysis of data collected as part of a randomised controlled trial testing a psychosocial intervention for antenatal depression. IPV, socio-demographic measures, depression and other mental health measures were collected at recruitment (first antenatal visit), 8 months gestation, and 3 and 12 months postpartum. IPV was defined as a sexual or physical violence perpetrated by the participant's partner in the past 3 months. Descriptive statistics are reported.

**Results.:**

Of 425 recruited depressed participants, 59 (13.9%) reported IPV at baseline, with physical IPV being the most frequently reported (69.5%). Reported IPV was associated with greater emotional distress, potentially higher food insecurity and higher rates of alcohol abuse. There were clear longitudinal trends in reported IPV with the majority of women no longer reporting IPV postpartum. However, some women reported IPV at later assessment points after not reporting IPV at baseline.

**Conclusions.:**

There is a strong association between IPV and depression in pregnancy. IPV reports remit over time for the women in this study, although the reason for this reduction is not clear and requires further investigation.

## Background

Depression and intimate partner violence (IPV) frequently co-occur (Melville *et al.* 2010; Connelly *et al.* 2013; Mitchell *et al.* 2016) and risk for both is known to be elevated in the antenatal and postnatal period (Beydoun *et al.* 2012; Howard *et al.* 2013). This co-occurrence during the perinatal period has important implications for maternal and child outcomes (Stein *et al.* 2014). Understanding this interaction is important to inform interventions addressing depression and IPV in pregnancy.

IPV includes physical, sexual or emotional violence perpetrated primarily by men on their female partners (Jewkes, 2002; Abrahams *et al.* 2006). In this paper, we focus on physical and sexual violence as verbal abuse usually coincides with either physical or sexual abuse (Mitchell *et al.* 2016) and the latter two are the most frequently investigated (Devries *et al.* 2013). IPV is a global phenomenon: a 2010 analysis of data from 81 countries on IPV against women showed that an estimated 30% of women 15 years and older reported a lifetime experience of IPV (Devries *et al.* 2013). This analysis showed important regional variations with women in sub-Saharan Africa (SSA) reporting the highest rates: 66% of women in central SSA and 42% of women in west SSA reported IPV. In southern SSA, 30% of women reported a lifetime experience of IPV (Devries *et al.* 2013). In 2003/2004 in South Africa, 29% of women reported being victims of IPV (Gass *et al.* 2011).

IPV increases the risk of depression (Lund *et al.* 2011; Fisher *et al.* 2012). Two recent systematic reviews of the effect of domestic violence (Howard *et al.* 2013) and IPV (Beydoun *et al.* 2012) on perinatal mental disorders show a strong association of severe depressive symptoms with experiences of partner violence while pregnant. Violence experienced postpartum results in a threefold increase in the likelihood of high postnatal depressive symptoms compared with women who do not experience IPV postpartum (Howard *et al.* 2013). Furthermore, the longer the duration of the exposure to abuse, the more pronounced the impact on depressive symptoms (Bonomi *et al.* 2006) in high-, middle- and low-income countries.

Independent of IPV, depression is a recognised feature of the perinatal period with estimates varying across different contexts. A systematic review of perinatal depression among African women provided a weighted mean prevalence of 11.3% [95% confidence interval (CI) 9.5–13.1%] for antenatal depression and 18.3% (95% CI 17.6–19.1%) for postnatal depression (Sawyer *et al.* 2010). However, in a study undertaken in the low-income context of Khayelitsha in Cape Town, South Africa, 39% of pregnant women reported depressive symptoms, with the main predictors of these symptoms being IPV and lack of support from partners, low household income and being younger in age (Hartley *et al.* 2011). In turn, perinatal depression has been shown to carry a variety of negative consequences for infant growth and development (Hayes & Sharif, 2009; Wachs *et al.* 2009; Stein *et al.* 2014). Psychosocial interventions for perinatal depression aimed at giving women coping, and problem solving skills have been shown to be effective (Rahman & Creed, 2007; Rahman *et al.* 2008; Chowdhary *et al.* 2013; Dennis & Dowswell, 2013; Singla *et al.* 2014).

However, there is a dearth of research evidence on the course of perinatal IPV, especially in low-resource settings in SSA. An understanding of the nature of the longitudinal relationship between IPV and perinatal depression can inform psychosocial interventions to address both problems.

Given this gap on the longitudinal pattern of reported IPV during the perinatal period, we present a secondary analysis of the Africa Focus on Intervention Research for Mental Health – South Africa (AFFIRM-SA) randomised controlled trial (RCT) data (Lund *et al.* 2014). The AFFIRM-SA RCT provided a psychosocial counselling intervention for pregnant women showing depressive symptoms. One of the secondary outcomes measured was the presence of IPV. These longitudinal data provide an opportunity to describe the progression of IPV over the perinatal period, in relation to depression and other health, social and psychological factors.

This paper aims to explore the prevalence, incidence and correlates of IPV among a group of pregnant and postnatal women with depressive symptoms in a low-income setting, and to compare child and maternal clinical outcomes at 3 months postpartum between women who experience IPV during pregnancy and those who do not.

## Methods

### Setting

This study reports descriptive secondary data collected as part of the AFFIRM-SA RCT, testing the effectiveness of a psychosocial counselling intervention for pregnant women with depressive symptoms. The RCT took place in Khayelitsha, a peri-urban informal settlement on the outskirts of Cape Town, South Africa. Information about recruitment, selection of instruments and data collection procedures for the RCT was published previously (Lund *et al.* 2014, 2015). The assessors at each time point were blind as to the arm allocation of the participant.

### Participants

Women were recruited by trained fieldworkers when attending their first antenatal clinic check-up at two midwife obstetric units. These women were at least 18 years of age, lived in Khayelitsha, spoke isiXhosa and were not more than 28 weeks pregnant at the time of recruitment. The participants were recruited based on their score on the screening tool – the Edinburgh Postnatal Depression Scale (EPDS) (Cox *et al.* 1987; Gibson *et al.* 2009). Depressive symptoms were defined as a score of 13 or higher on the EPDS. A positive screening score resulted in recruitment of that participant. This was followed by a full baseline assessment on the same day of recruitment by the same fieldworker. The fieldworkers were not involved in the medical care of the women and worked independently of the services provided by the midwives at the antenatal clinic. The screening and baseline assessments were conducted in a quiet room away from the main waiting area for the clinic. The nature of the public health services in this context are such that women spend 2–4 h waiting to be seen. This gave the fieldworkers a good opportunity to screen and assess the women.

The full sample enrolled in the AFFIRM-SA trial (*n* = 425) were included in the analysis for this paper. If participants reported IPV, they were encouraged to report it to their local police station. If a woman reported suicidal ideation or behaviour, she was referred to the clinic psychiatric nurse for further assessment and management.

### Measurement instruments

A range of socio-demographic, socio-economic and clinical measures were collected at four time points during the perinatal period: at recruitment (baseline), 8 months gestation, 3 months postpartum and 12 months postpartum. The measures collected at all assessment time points are described in the AFFIRM-SA RCT protocol (Lund *et al.* 2014). Briefly, these included the AFFIRM Functional Assessment Instrument (AFFIRM-FAI) (Schneider *et al.* 2015), the WHO Disability Assessment Schedule 2.0 (12 items) (WHODAS 2.0) (Üstün *et al.* 2010), the Multidimensional Scale of Perceived Social Support (MSPSS) (Zimet *et al.* 1988), the WHO Alcohol Use Disorder Identification Test (AUDIT) (Saunders *et al.* 1993) and the Household Food Insecurity Access Scale (HFIAS) (Coates *et al.* 2006). The Hamilton Depression Rating Scale (HDRS; Hamilton, 1960) was used to provide a more robust measure of depressive symptoms as compared with the EPDS. For this study, an adapted version of the HDRS was created for lay interviewers, based on the adapted version of the HDRS by Potts *et al.* (1990) and Williams (1988). Birth and child outcomes were measured at the two postnatal assessment points, and these included Apgar scores, infant head circumference, weight and height, and incidence of respiratory and diarrhoeal disease.

Reports of IPV at each assessment time point were recorded by asking the participants whether they had been (1) the victim of physical violence in the last 3 months (e.g. beating, pushing, kicking, biting, slapping, etc.); and (2) the victim of sexual violence in the last 3 months. If participants answered affirmatively to one or both questions, they were asked by whom. Physical or sexual IPV was defined as responding yes to the first or second question, respectively, and specifying the violence was perpetrated by the partner. As stated earlier, we did not include verbal abuse as it usually coincides with either physical or sexual abuse (Mitchell *et al.* 2016) and the latter two are the most frequently investigated (Devries *et al.* 2013).

### Data analysis

Data collected as part of the assessments were captured on an android mobile device system (Mobenzi; https://www.mobenzi.com), which minimised missing data for completed assessments. Attrition at follow-up ranged between 21 and 29%, and was highest at the 8-month gestation assessment. The main reasons for missed assessments were because of having given birth (for 8-month gestation assessment), not being contactable or refusal to continue their participation. Participants were suspended from the trial if they had a miscarriage or stillbirth, but were offered continued counselling outside of the trial intervention.

Once data collection was complete, these were transferred to Stata 13 for analysis.

The preliminary (as yet unpublished) results of the RCT suggest that the intervention and control arms showed little difference in depressive symptoms at 3 months postpartum, the primary outcome measure. For this reason, the two arms were combined and analysed as one group for the purpose of this study. No imputation was conducted to address missing assessments.

Given the small sample of women reporting different forms of IPV at baseline, physical and sexual IPV were analysed together and only descriptive analyses and non-parametric univariate analyses were conducted. The Fisher's exact test was used to assess baseline categorical variables in relation to IPV at baseline or the incidence of IPV later in pregnancy or postpartum. The same test was used to compare categorical birth, infant and maternal outcomes at 3 months postpartum between participants reporting IPV during pregnancy and those who did not. Mann–Whitney *U* tests were performed to assess continuous correlates of IPV at baseline and continuous infant and maternal outcomes at 3 months postpartum. Correlates of the incidence of IPV later in pregnancy or postpartum *v.* those who never reported IPV were investigated using the Kruskal–Wallis test.

The child and maternal outcomes assessed at 12 months postnatal assessment were not included in these analyses, since we could not control for potential confounders.

## Results

### Sample characteristics and the prevalence of IPV at baseline

Out of the 425 participants, 59 participants (13.9%) reported having experienced physical and/or sexual IPV in the past 3 months at baseline: 52 (12.2%) reported physical IPV and 18 (4.2%) reported sexual IPV. Altogether, 11 (2.6%) participants reported experiencing both forms of IPV. Table 1 presents the baseline demographic and clinical characteristics of the women recruited into the study. The majority of the participants were between 22 and 30 years of age (*n* = 235, 55.3%) with a mean age of 27.3 years (s.d. = 5.72). Nearly all women reported having a partner (*n* = 423, 99.5%), but only a third actually lived with their partner (34.8%). Approximately 40% of the sample had finished secondary education, and nearly half were employed at the time of recruitment (*n* = 193, 45.4%). Participants in the study all had symptoms of depression based on the EPDS (as per the inclusion criteria). However, 41.4% were diagnosed with clinical depression on the MINI, and 17.4% were considered at high risk of suicide.

**Table 1.** Demographic and clinical characteristics of sample at recruitment (baseline)

Demographic and clinical variables	Total (*N* = 425)		No IPV (*N* = 366)		IPV (*N* = 59)		χ^2^
	*N*	%	*N*	%	*N*	%	
Age							1.52
18–21 years	74	17.4	65	17.8	9	15.2	
22–30 years	235	55.3	205	56.0	30	50.9	
31 years or more	116	27.3	96	26.2	20	33.9	
Marital status							6.20*
Lives with a partner	148	34.8	119	32.5	29	49.2	
Does not live with a partner	277	65.2	247	67.5	30	50.8	
Educational level							3.08^a^
Grade 0–11	251	59.1	210	57.4	41	69.5	
Grade 12 or more	174	40.9	156	42.6	18	30.5	
Employment							2.15
Employed	193	45.4	161	44.0	32	54.2	
Unemployed/studying	232	54.6	205	56.0	27	45.8	
Food status							6.80^a^
Food secure	107	21.2	94	25.7	13	22.0	
Mildly food insecure	97	22.8	90	24.6	7	11.9	
Moderately food insecure	94	22.1	76	20.8	18	30.5	
Severely food insecure	127	29.9	106	29.0	21	35.6	
HIV status							1.88
Negative	282	68.6	248	69.9	34	60.7	
Positive	129	31.4	107	30.1	22	39.3	
MINI diagnosis							14.93***
Not depressed	249	58.6	228	62.3	21	35.6	
Depressed	176	41.4	138	31.7	38	64.4	
Suicide risk							6.19*
Low	351	82.6	309	84.4	42	71.2	
High	74	17.4	57	15.6	17	28.8	
	Median	IQR	Median	IRQ	Median	IQR	*U*
HAM-D score	15	6	15	6	18	7	−2.95*
EPDS score	17	5	17	5	18	7	−2.96**
WHODAS score	25	25	25	25	22.2	25	1.08
AUDIT score	0	6	0	5	2	11	−2.72**
MSPSS score	60	16	60	16	61	24	0.66

*Significant at <0.05 level; **significant at <0.01 level; ***significant at <0.001 level.

a Marginal.

### Comparison of clinical and socio-demographic characteristics of participants with and without IPV reports at baseline

Table 1 further presents the comparison of demographic and clinical characteristics of participants at baseline, depending on whether they report experiencing IPV or not. According to the Fisher's exact test analyses, a greater proportion of participants reporting IPV lived with their partner (49.2%), compared with those who did not report IPV (32.5%) (χ^2^ = 6.20, *p* < 0.05). There were no other demographic differences between the two groups, though a marginally greater proportion of those who reported IPV had a lower education level and greater food insecurity.

Clinically, however, participants who experienced IPV at baseline had significantly worse symptoms (i.e. higher scores) on the HAM-D (median = 18, IQR = 7; *U* = −2.95, *p* < 0.05), EPDS (median = 18, IQR = 7; *U* = −2.96, *p* < 0.01) and the AUDIT (median = 2, IQR = 11; *U* = −2.72, *p* < 0.01), compared with participants who did not report IPV (HAM-D: median = 15, IQR = 6; EPDS: median = 17, IQR = 5; AUDIT: median = 0, IQR = 5). Finally, as measured by the MINI, nearly twice as many participants who reported IPV received a diagnosis of depression (64.4%; χ^2^ = 14.93, *p* < 0.001) and were at a significantly high risk of suicide (28.8%; χ^2^ = 6.19, *p* < 0.05), compared with participants not experiencing IPV (31.7 and 15.6% for depression and suicide risk, respectively). The two groups did not differ on levels of impaired functioning or on their perceived level of social support.

### Change in IPV reports over time

Of participants who reported IPV at baseline, the majority no longer reported experiencing IPV at 3 months post-partum (*n* = 43, 72.9%). Of these, one reported no longer being with a partner at the 8-month gestation assessment, and a further 10 reported no longer being with a partner at the time of the 3-month postpartum assessment. This suggests that the reported IPV subsided before the person separated from their partner. Over a quarter of participants (*n* = 16, 27.1%), however, continued to experience IPV during the postpartum period. Of those who did not experience IPV at baseline (*n* = 366, 86.1%), 12.8% (*n* = 47) went on to report IPV in later assessments, either later in pregnancy (*n* = 14, 3.8%) or in the postpartum period (*n* = 33, 9.0%).

### Correlates of the incidence of IPV later in pregnancy or postpartum

Table 2 presents the results of the Fisher's exact and Kruskal–Wallis tests to identify correlates of the IPV incidence in late pregnancy (8 months gestation) or postpartum (3 and/or 12 months postpartum). These two groups were also compared with participants who never reported IPV during the course of the study (*n* = 319, 75.1%). Results suggest that a greater proportion of participants who lived with their partners at baseline reported IPV starting at the end of pregnancy (8.4%), compared with those who did not live with their partner (1.6%) (χ^2^ = 12.23, *p* < 0.01). A greater proportion of participants who were employed at baseline reported the incidence of IPV at the end of pregnancy (6.2%), rather than in the postnatal period (5.2%) (χ^2^ = 6.79; *p* < 0.05). The opposite was true for those who were unemployed at baseline, with 2.0% reporting the incidence of IPV late in pregnancy, and 11.2% postpartum (χ^2^ = 6.79, *p* < 0.05). Age was also marginally associated with the incidence of IPV: older participants (aged 31 years or more) were more likely to experience IPV later in pregnancy. None of the younger participants (aged 18–21) reported IPV before birth, but the incidence of IPV postpartum was highest in this younger age group.

**Table 2.** Differences in demographic and clinical characteristics between participants who never reported IPV and those who did not report IPV at baseline but report it later in pregnancy or postnatally^a^

Demographic and clinical variables	*N*	No perinatal IPV (*N* = 319)		Late antenatal incidence of IPV (*N* = 14)		Postnatal incidence of IPV (*N* = 33)		χ^2^*H*
		*N*	%	*N*	%	*N*	%	
Age								8.34^b^
18–21 years	65	56	86.2	0	0	9	13.8	
22–30 years	205	184	89.8	8	3.9	13	6.34	
31 years or more	96	79	82.3	6	6.2	11	11.5	
Marital status								12.23**
Lives with a partner	119	95	79.8	10	8.4	14	11.8	
Does not live with a partner	247	224	90.7	4	1.6	19	7.7	
Educational level								0.47
Grade 0–11	210	181	86.2	9	4.3	20	9.5	
Grade 12 or more	156	138	88.5	5	3.2	13	8.3	
Employment								6.79*
Employed	161	141	87.6	10	6.2	10	5.2	
Unemployed/studying	205	178	86.8	4	2.0	23	11.2	
Food status								6.56
Food secure	94	85	90.4	5	5.32	4	4.26	
Mildly food insecure	90	79	87.8	4	4.4	7	7.8	
Moderately food insecure	76	66	84.8	1	1.3	9	11.8	
Severely food insecure	106	89	84.0	4	3.8	13	12.2	
HIV status								1.43
Negative	248	220	88.7	8	2.2	20	8.1	
Positive	107	90	84.1	5	4.7	12	11.2	
MINI diagnosis								4.90^b^
Not depressed	228	204	89.5	5	2.2	19	8.3	
Depressed	138	115	83.3	9	6.5	14	10.1	
Suicide risk								1.57
Low	309	270	87.4	13	4.2	26	8.4	
High	57	49	86.0	1	1.8	7	12.3	
	*N*	Median	IQR	Median	IQR	Median	IQR	*H*
HAM-D score	366	15.0	6.0	13.5	8.0	17.0	8.0	5.50^b^
EPDS score	366	16.0	5.0	16.5	5.0	18.0	5.0	3.44
WHODAS score	366	25.0	25.0	16.7	22.2	33.3	33.3	4.72^b^
AUDIT score	366	0	5.0	0	4.0	6	13.0	12.34**
MSPSS score	366	61.0	15.0	57.0	21.0	58.0	20.0	2.23

a Percentages presented as rows; *significant at <0.05 level; **significant at <0.01 level.

b Marginal.

Participants who reported postnatal incidence of IPV had significantly greater AUDIT scores at baseline (median = 6.0, IQR = 13.0; *H* = 12.34, *p* < 0.01), compared with the other two groups (no IPV: median = 0, IQR = 5.0; late antenatal IPV: median = 0, IQR = 4.0). A similar, but marginal trend was found for HAM-D scores: participants who reported IPV postpartum had marginally greater baseline HAM-D scores compared with those who never reported IPV, or those who reported the incidence of IPV later in pregnancy. Finally, a marginally greater proportion of women diagnosed with depression at baseline reported the incidence of IPV, both before and after birth (6.5 and 10.1%, respectively), compared with those who were not diagnosed with depression (2.2 and 8.3%, respectively).

### Birth, infant and maternal outcomes among women who report IPV during pregnancy

The birth and new-born outcomes were compared between participants who reported IPV during pregnancy (baseline and/or 8 months gestation) and those who did not (Table 3). No differences were found between the two groups in terms of birth, delivery or new-born outcomes. Marginal differences were found in Apgar scores, in that a greater proportion of babies of participants who reported IPV during pregnancy had a score of 10, compared with babies of participants who did not experience IPV during pregnancy.

**Table 3.** Comparison of delivery, birth and new born outcomes between participants who report IPV during pregnancy and those who do not

Outcomes	*N* (335)	No IPV during pregnancy (*N* = 281)		IPV during pregnancy (*N* = 54)		χ^2^
		*N*	%	*N*	%	
Delivery outcomes						
Timing of delivery						0.02
Preterm (live birth)	42	35	12.5	7	13.0	
At term	251	211	75.1	40	74.1	
Over term	42	35	12.5	7	13.0	
Length of labour						2.71
12 h or less	215	177	63.0	38	70.4	
13–24 h	71	59	21.0	12	22.2	
More than 24 h	49	45	16.0	4	7.4	
Type of delivery						1.92
Vaginal	234	192	68.3	42	77.8	
Caesarean	101	89	31.7	12	22.2	
Problematic delivery						<0.01
No	280	235	83.6	45	83.3	
Yes	55	46	16.4	9	16.7	
Time spent in hospital						1.25
Less than a day	123	104	37.8	19	35.8	
1–3 days	128	104	37.8	24	45.3	
More than 3 days	77	67	24.4	10	18.9	
New born outcomes						
Placed in incubator	30	28	10.0	2	3.7	2.18
Problems with infant's health	29	22	7.8	7	13.0	1.51
Apgar scores						3.31^a^
Below 10	61	56	22.3	5	10.6	
10	237	195	77.7	42	89.4	
	*N*	Median	IRQ	Median	IQR	*U*
Birthweight (kg)	323	3.1	0.6	3.1	0.9	−0.03
Birth height (cm)	306	49	4	49	3	−0.437
Head circumference at birth (cm)	306	34	2	34	3	0.362

a Marginal.

Table 4 illustrates differences in infant and maternal outcomes at 3 months postpartum, again between participants who reported IPV during pregnancy (baseline and/or 8 months gestation) and participants who never reported IPV during pregnancy. This time, however, participants who reported the incidence of IPV at 3 months postpartum were excluded from the analysis, so that any association between IPV during pregnancy and outcomes could not be confounded by current IPV.

**Table 4.** Comparison of child and maternal outcomes at 3 months postpartum between participants who report IPV during pregnancy and those who do not

Outcomes	*N* (281)	No IPV during pregnancy (*N* = 248)		IPV during pregnancy (*N* = 33)		χ^2^ or *U*
		*N* or median	% or IQR	*N* or median	% or IQR	
Child outcomes						
Weight (kg)	272	6.0	1.2	6.1	1.6	−0.52
Height (cm)	248	59.0	7.0	59.0	7.0	0.36
Head circumference (cm)	247	42.0	4.0	41.0	3.0	1.23
Duration of breastfeeding (weeks)	281	17.0	7.0	17.0	11.0	0.96
Suffered from (in past 2 weeks):						
Diarrhoea	61	54	21.8	7	21.2	0.01
Difficulty breathing	76	67	27.0	9	27.3	<0.01
Cough	138	121	48.8	17	51.5	0.09
Maternal outcomes						
EPDS score	281	7.0	7.0	10.0	8.0	−2.59*
HAM-D score	281	8.0	6.0	11.0	4.0	−2.84*
WHODAS score	281	13.9	19.4	13.9	25.0	−1.17
AUDIT score	281	0	2.0	0	4.0	−1.40
Depression diagnosis						1.15
Not depressed	239	213	85.9	26	78.8	
Depressed	42	35	14.11	7	21.2	

*Significant at <0.05 level.

No differences were found between the two groups, either in terms of anthropometric characteristics, breastfeeding length or health problems. Differences were found in relation to maternal clinical symptoms, however. Participants who reported IPV during pregnancy had greater depressive symptoms at 3 months postpartum, as measured by the HAM-D (median = 11.0, IQR = 4.0, *U* = −2.84, *p* < 0.05) and the EPDS (median = 10.0, IQR = 8.0, *U* = −2.59, *p* < 0.05), compared with those who never reported IPV (HAM-D: median = 8.0, IQR = 6.0; EPDS: median = 7.0, IQR = 7.0).

*Post hoc* analyses were conducted to identify whether these results remained the same, after controlling for baseline depressive symptoms (not reported in Table 4). Among participants not diagnosed with depression at baseline, those who reported IPV during pregnancy had significantly higher HAM-D scores at 3 months postpartum (median = 8.0; IQR = 6.0). Non-significant trends also suggest that among those not diagnosed with depression at baseline, a greater proportion of women were diagnosed with depression at 3 months postpartum, compared with those who did not report IPV during pregnancy.

## Discussion

This paper used longitudinal data to explore the prevalence, incidence and correlates of IPV among a group of pregnant and postnatal women with depressive symptoms in a low-income setting, and to compare child and maternal clinical outcomes at 3 months postpartum between women who experience IPV during pregnancy and those who do not. The results of our analysis showed that the participants in the AFFIRM-SA trial did report both IPV and depression and that those women who reported IPV were more likely to also be depressed. This confirms the common co-occurrence found in other studies both local and more global (Melville *et al.* 2010; Connelly *et al.* 2013; Mitchell *et al.* 2016). However, the AFFIRM-SA trial did not show any significant effect of this co-occurrence on child outcomes for the measures used in the trial.

At the baseline assessment, the prevalence of any type of sexual or physical IPV in the preceding 3 months was reported by 13.9% (*n* = 59) of the sample of pregnant women with depressive symptoms, with the majority of these being physical IPV. Limited comparisons can be made between the prevalence of IPV reported in this study to that of other studies, as most studies investigated trends among all pregnant women. However, there is evidence that pregnant women who are depressed report higher rates of IPV than those not depressed when screening all pregnant women. Connelly *et al.* (2013) found that 10.2% of pregnant women screening positive on the EPDS reported physical and/or sexual IPV compared with only 1.8% who screened negative. Melville *et al.* (2010) report similar findings for physical and/or sexual IPV reports for pregnant women who had ‘probable major depression’ as measured using the Patient Health Questionnaire, with 20.8% reporting IPV compared with only 2.9% without depression. The rate of 13.9% found in our study confirms this strong association of IPV and depression, but cannot confirm the comparison with reports of IPV by pregnant women without depression.

The results show that 17.4% of all participants were deemed at high risk of suicide because of reported suicidal ideation and/or behaviour. However, women who reported IPV were twice more likely to be at risk for suicide than those without IPV. These findings resonate with those from other studies. In an epidemiological review of studies, prevalence of suicidal ideation in pregnancy (including non-depressed women) range from 5 to 20% in low- and middle-income contexts (Gelaye, Kajeepeta & Williams, 2016). The AFFIRM-SA study focused only on depressed pregnant women and hence the rate of 17.4% for suicidal ideation does not represent a rate for the broader depressed and non-depressed population of pregnant women. As we were not able to find a similar study identifying suicidal ideation rates for depressed pregnant women specifically, it is difficult to compare the AFFIRM-SA rates with existing rates. Pregnant women with suicidal ideation are generally more likely to report IPV than those without suicidal ideation (Gavin *et al.* 2011; Gelaye *et al.* 2016). Hence, the results from the AFFIRM-SA trial show high but not unexpected rates of suicidal ideation and show the same patterns in relation to reported IPV.

When considering the longitudinal nature of the results, we find some notable trends. There was a marked reduction in reports of partner abuse after the baseline assessment overall. Only a minority of participants who no longer reported IPV after baseline were no longer with their partner. For those women who report having a partner but no longer report IPV, the data do not indicate if this is the same partner or a different partner from the person who was reported as the perpetrator of IPV. While IPV subsides among those who report IPV at baseline, there seems to be a surge of women reporting the incidence of IPV after giving birth.

While there were few notable differences in demographic factors between women reporting and not reporting IPV, there were some distinct differences in clinical characteristics: women who experience IPV have more severe depressive symptoms, are at greater risk of suicide and are more likely to have a diagnosis of depression. They are also more likely to abuse alcohol. These findings reflect trends noted in other studies (Ludermir *et al.* 2010; Melville *et al.* 2010; Connelly *et al.* 2013; World Health Organization, 2014). These findings highlight the need to sensitise health care providers to identify risk factors, including IPV and substance use, as suggested by Connelly *et al.* (2013), and to use antenatal services as a good location for identifying women at risk, as suggested by the WHO (2014).

There was a strong association between IPV and depression, and our study provides evidence for a bi-directional relationship between the two: depressive symptoms were (marginal) predictors of IPV later in pregnancy and postpartum, and IPV during pregnancy predicted high depressive symptoms at 3 months postpartum. However, the small sample and non-parametric analyses mean that we need to be cautious about these findings, as the small sample size did not allow for multivariate analyses, and so we could not control for any confounders.

There were no differences in birth, new-born or infant outcomes between participants reporting IPV *v.* no IPV during pregnancy. This could be due to the small sample and the lack of variation across these measures in the overall sample. It could also be explained in part by the nature of the cohort, in that all participants were already compromised because of their depressive symptoms, which themselves are associated with adverse birth outcomes (Stein *et al.* 2014). Shah & Shah (2010) concluded from their systematic review that low birth weight and preterm delivery were significant outcomes of domestic violence reported during pregnancy. However, they also hypothesise that there is under-reporting of domestic violence as their sample was small.

The two IPV groups (late pregnancy *v.* postnatal) do not differ much in terms of demographic characteristics. They do, however, differ in terms of depressive symptoms and alcohol use behaviours: participants who report IPV starting postpartum had greater depressive and alcohol abuse symptoms at baseline than participants who report IPV during the course of pregnancy. The reasons for this are not clear and could be elucidated through further research more specifically targeted at IPV in the perinatal period and specifically following up women postpartum.

The results do suggest, however, that women who experience IPV during pregnancy are more likely to have greater depressive symptoms at 3 months postpartum. This was also the case among women who were not diagnosed with depression at baseline (though showed some symptoms of depression – as per inclusion criteria). This suggests that IPV may have been present before worsening of depressive symptoms.

The profile of the participants reporting any IPV in our study reflect a high degree of vulnerability, with greater emotional distress, a tendency towards higher food insecurity and higher rates of alcohol abuse, compared with women without IPV. These concur with profiles reported in other studies, with food insecurity and unemployment being common aspects associated with the reports of IPV (Bonomi *et al.* 2006; Malan, 2017).

These profiles of high vulnerability did give an indication as to the direction of causality, as these were present before any report of IPV suggesting these factors may be predisposing factors for IPV. Further research is needed with a bigger sample to investigate these causal pathways further, bearing in mind that IPV reported at baseline was for the 3 months prior to the assessment, and may have been present before that.

While we are not able to conclude on the effectiveness of the AFFIRM-SA intervention on targeting IPV perpetration and victimisation, future research could consider combining a psychosocial intervention with an IPV prevention or mitigation intervention.

## Limitations

This paper gives a broad overview of the context of IPV in relation to depression in pregnancy and other related clinical and socio-demographic factors. The limited sample did not allow for more complex statistical analyses to be conducted to determine interactions among identified correlates of IPV at baseline or later in pregnancy and postpartum. This is largely due to reported IPV being a secondary outcome of the trial and hence not considered in the power calculations for the original sample.

The data analysed are from a selected cohort of pregnant women with depressive symptoms. Reporting of IPV for all women regardless of depression symptoms may have highlighted other possible explanations for the association between IPV and depression and provided a prevalence estimate for the larger population. An IPV-focussed study would provide more evidence on the validity of trends noted in the AFFIRM-SA data and possibly highlight other trends such as positive associations with functioning and perceived social support identified in other studies (Bonomi *et al.* 2006; Malan, 2017). Such a study could also provide information on verbal and emotional abuse in addition to the physical and sexual IPV addressed in the AFFIRM-SA study.

Lastly, the focus of the AFFIRM-SA trial was on prenatal depression and hence focussed on measuring depression rather than a broader spectrum of mental disorders. Further research should consider other common mental disorders such as anxiety and post-traumatic stress disorders to provide a more comprehensive perspective of the association of IPV with mental disorders.

## Conclusions

This study found that experiences of IPV among antenatal women with depressive symptoms were associated with greater emotional distress, potentially higher food insecurity and higher rates of alcohol abuse. These factors describe profiles for women reporting IPV and suggest situations of high vulnerability. Trends noted in the AFFIRM-SA findings are similar to findings from other studies across a wide range of countries that highlight the strong correlation between pregnancy, depression and IPV.

## Ethical considerations

The AFFIRM-SA trial was approved by the Human Research Ethics Committee of the University of Cape Town (ref number: 226/2011) and by the Western Cape Department of Health and health facilities involved in the trial. Each participant signed an informed consent form before being recruited into the study.
